# Effectiveness of Prophylactic Bolus Ephedrine Versus Norepinephrine for Management of Postspinal Hypotension during Elective Caesarean Section in Resource Limited Setting: A Prospective Cohort Study

**DOI:** 10.1155/2022/7170301

**Published:** 2022-10-03

**Authors:** Mitiku Desalegn, Tewoderos Shitemaw, Habtamu Tamrat

**Affiliations:** ^1^Department of Anesthesia, Wachemo University, College of Medicine and Health Science, Hossana, Ethiopia; ^2^Department of Anesthesia, Menelik II College of Medical & Health Science, Kotebe Metropolitan University, Addis Ababa, Ethiopia; ^3^Department of Orthopedics, College of Medicine and Health Science, Wachemo University, Hosanna, Ethiopia

## Abstract

**Background:**

Spinal anaesthesia for caesarean section is the preferred technique since it provides better maternal safety and neonatal outcome compared to general anaesthesia. Hypotension is the most common complication after spinal anaesthesia. The study aims to determine the effectiveness of a prophylactic bolus dose of norepinephrine and ephedrine on the management of postspinal hypotension during caesarean section.

**Method:**

An institutional-based prospective cohort study was conducted on 84 pregnant women undergoing elective caesarean section. Based on the responsible anaesthetist's postspinal hypotension management plan, patients were divided into two groups. Those patients who received ephedrine are grouped into the ephedrine (EPH, *n* = 42) group, and patients who received norepinephrine are grouped under the norepinephrine group (NE, *n* = 42) by data collectors. After aseptic technique, spinal anaesthesia was administered with 0.5% (3 ml) bupivacaine using a 23G spinal needle. During spinal anaesthesia, a prophylactic bolus dose of 10 mg (2 ml) EPH or 16 g (2 ml) NE was given based on management plan of the shift anaesthetist. Mean arterial pressure (MAP), the heart rate (HR), number of boluses of vasopressor used, incidence of nausea and vomiting, and the Apgar score of babies at 1 and 5 min between the groups were recorded.

**Results:**

The norepinephrine group had a statistically significant higher MAP compared to the ephedrine group in the first 10 and 15 min (*p* < 0.05) of the study period. Thereafter, there was no statistically significant difference in MAP between the groups until the end of the study period (*p* > 0.05). The ephedrine group had a statistically significant higher heart rate throughout the procedure compared to the norepinephrine group (*p* < 0.05). The norepinephrine group required a lower bolus number of vasopressors compared to the ephedrine group to maintain blood pressure. The Apgar scores of all babies at 1 and 5 min were above seven. Significant differences regarding maternal complications (nausea and vomiting) between the groups were not detected (nausea, *p*=0.21 and vomiting, *p*=0.092).

**Conclusion:**

Norepinephrine can be used instead of ephedrine to keep a pregnant mother's blood pressure stable during a caesarean section under spinal anaesthesia without causing harm to the mother or baby. Trial registration. ClinicalTrials.gov Identifier: NCT05522088 (Date of registration: 30/08/22).

## 1. Introduction

In situations where spontaneous vaginal delivery is a threat to the mother and baby, caesarean section (CS) is preferred to save lives [1]. The acceptance of spinal anaesthesia for CS has increased extensively since it provides better maternal safety and neonatal outcome compared to general anaesthesia [2]. However, spinal anaesthesia for CS is not without side effects. Hypotension (a decrease in systolic blood pressure below 80% of baseline) is the most frequent complication, with an incidence ranging from 7.4% to 74.4% [3]. Dominance of vasodilation over vasoconstriction is the most common mechanism underlying hypotension associated with spinal anaesthesia. The mechanism for vasodilatation after spinal anaesthesia is due to blockade of the sympathetic nerve fibers at the preganglionic level [4].

Evidence shows that intraoperative hypotension is independently associated with postoperative 30 day mortality, major adverse cardiac events (MACEs), especially myocardial injury, and acute kidney injury (AKI) in adult patients after noncardiac surgery [5]. Another study found an increased risk of end-organ injury during prolonged (≥10-minute) exposure to mean arterial blood pressure (MAP) < 80 mm Hg and for shorter durations < 70 mmHg during the intraoperative period [6].

A range of approaches are used to prevent postspinal hypotension, such as leg compression, preloading with crystalloid or colloids, coloading with crystalloids, and the use of a variety of vasopressors [7]. The recent “International consensus statement on the management of postspinal hypotension” recommends the use of vasopressors [8]. EPH is one of the most widely used vasopressors used for management of postspinal anaesthesia for parturients undergoing caesarean section [9]. Its main mode of action is attained indirectly by inhibiting neuronal norepinephrine reuptake into preganglionic neurons, which increases the amount into the synapse [10]. Administration of NE for postspinal hypotension for patients undergoing caesarean section is common and effective in maintaining blood pressure. NE is a sympathomimetic drug; it primarily has agonistic action at alpha1 and beta1 receptors, with little-to-no beta2 or alpha2 activity [11].

Evidence shows that the occurrence of intraoperative hypotension is associated with multiple intraoperative and postoperative complications. The use of fluid for the prevention of postspinal hypotension is unsatisfactory for many anaesthetists. EPH and NE are the widely practiced and accepted choices of vasopressors for management of postspinal hypotension during caesarean section. However, optimum bolus dosing is not yet established. Automated infusion systems are recommended for administration of measured doses of drugs, but their accessibility in developing countries is low so that establishment of an optimum bolus dose of vasopressor is warranted. Hence, the primary outcome of this study is to compare MAP between EPH and NE groups. The secondary outcomes are comparing the bolus number of vasopressors required, HR, and Apgar scores between the two groups.

## 2. Method and Material

### 2.1. The Study Design and Setting

An institutional-based prospective observational cohort study was conducted from March 01, 2022 to April 30, 2022 at Wachemo University Nigist Eleni Mohamed Memorial comprehensive specialized hospital after obtaining approval from the local ethical committee. Informed written consent was obtained from each pregnant woman. This study was performed in accordance with the Declaration of Helsinki. Confidentiality was assured throughout the research.

American Society of Anaesthesiologist (ASA) class II and ages ranging from 18 to 35 years were included in the study. We excluded pregnant women with preeclampsia/eclampsia, baseline hypertension (systolic blood pressure > 140 mmHg), body mass index (BMI) > 30 kg/m^2^, failed spinal, spinal anaesthesia converted to general anaesthesia, contraindication for spinal anaesthesia, and mother with cardiovascular, renal, or hepatic disease.

### 2.2. Sample Size

The sample size was calculated by Open Epi version 7 by adjusting a power of 80%, confidence level of 95%, and margin of error of 5%. The primary outcome variable of our study is to compare MAP between the groups which was estimated from the previous study conducted by Elnabtity and Selim [12]. The minimum sample size of 42 patients per group (total 84 patients) was determined.

### 2.3. Preoperative and Intraoperative Procedure

The decision to administer vasopressor prophylaxis prior to spinal anaesthesia and the management of anaesthesia, including intraoperative treatment of hypotension, were made at the discretion of the anaesthetist assigned to each case. We, the researchers, were not involved in the perioperative management of the patients. Since the study site did not allow RCT, randomization was not employed rather patients were divided into EPH (*n* = 42) and NE (*n* = 42) groups based on the independent decision of the responsible anaesthetists. Those patients who received 10 mg (2 ml) of prophylactic EPH during induction of spinal anaesthesia were considered as the EPH group, and patients who received 16 *μ*g (2 ml) of prophylactic NE were considered as the NE group by the assigned data collector. We hypothesized that administering prophylactic NE during spinal anaesthesia for elective CS would better maintain maternal MAP compared to prophylactic EPH without having adverse effects on the mother or the baby.

Ephedrine sulphate(EPH) ampoules (50 mg in 2 ml; product of “Misr Company for Pharmaceuticals,” Egypt), diluted in 10 ml of normal saline (5 mg/ml) and norepinephrine ampules(NE) (4 mg in 4 ml, product of “Egy-Pharma Company,” Cairo, Egypt), diluted in 500 ml of normal saline (8 *μ*g/ml) were the usual preparation used in the setup.

The study site follows the following routine procedure during CS under spinal anaesthesia.

All patients were assessed before the procedure with a history, clinical examination, and routine laboratory investigation (complete blood count, liver function, renal function, and coagulation profile), and baseline blood pressure with a noninvasive blood pressure monitor and heart rate with pulse oximetry were recorded. On arrival to the operating room, two 18 gauge IV cannula needles are secured on bilateral arms. Patients were premedicated with metoclopramide 10 mg IV bolus before induction of anaesthesia and preloaded with normal saline (NS) at 10 ml/kg 20 minutes before the spinal anaesthesia. After aseptic technique, spinal anaesthesia was administered in a sitting position at the level of L2/L3 by a shift anaesthetist with 0.5% (3 ml) bupivacaine using a 23G spinal needle. Thereafter, patients were immediately turned to a supine position with slight head elevation using a pillow. The sensory and motor blocks were evaluated by a sense of coldness and the Modified Bromage Scale (0 = no motor block, 1 = inability to raise an extended leg, 2 = inability to flex the knee, and 3 = inability to flex the ankle and foot), respectively, within 5 min of intrathecal injection. During spinal anaesthesia, a prophylactic bolus dose of 10 mg (2 ml) EPH or 16 *μ*g (2 ml) NE was given based on the preference of the shift anaesthetist.

After providing training for data collectors, data were collected using pretested questionnaires. The data collectors assigned each selected participant to either group depending on the responsible anaesthetists' treatment strategy (whether they received prophylactic ephedrine or norepinephrine). This continues until the desired sample was obtained for each group.

MAP, HR, the number of boluses of vasopressor used and Apgar score at 1 and 5 min in each group were recorded. The incidence of nausea and vomiting and episodes of bradycardia (HR < 60 bpm) were recorded and informed to the assigned anaesthetist for treatment. MAP and HR were recorded every 5 minutes, up to 20 minutes, every 10 minutes till the end of surgery.

For the purpose of this study, some terms are defined in the following way.

#### 2.3.1. Baseline Value

Measurement taken before spinal anaesthesia is being administered.

#### 2.3.2. Hypotension

It is defined as a decrease in SBP ≥ 20% of baseline value.

#### 2.3.3. Postspinal Hypotension

Hypotension that occurs after administration of spinal anaesthesia.

#### 2.3.4. Sensory Block

This was defined as the inability to sense pinprick sensation at T-10 (umbilicus).

#### 2.3.5. Motor Block

This was defined as the inability to move hip, knees, or toes.

### 2.4. Statistical Analysis

Using SPSS software version 20.0, quantitative data were described in terms of mean SD for parametric and median (interquartile range) for nonparametric data. Frequency and percentage were used to describe categorical data. The Shapiro–Wilk test was used to test for normal distributions of data, while equality of variance between the groups was assessed using Levene's test. Comparisons of quantitative data between the study groups were carried out using an unpaired Student's *t*-test for parametric data and the Mann–Whitney *U* test for nonparametric data. The incidence and timing of hypotension occurrences were further analyzed using Kaplan–Meier survival analysis, with comparison between the groups using the log-rank test. The confidence interval was set to 95%, and the margin of error accepted was set to 5%. The significance level was determined at *p* value 0.05.

## 3. Result

A total of 84 pregnant women were enrolled in the study that fulfilled inclusion criteria and were randomly divided into two groups of 42 patients in each: the EPH group and the NE group. There is no statistically significant difference in demographic and intraoperative data between the two groups. Levels of block between the groups were comparable (T6-T8) (Table 1).

### 3.1. Haemodynamic Variables

**Figure 1:**
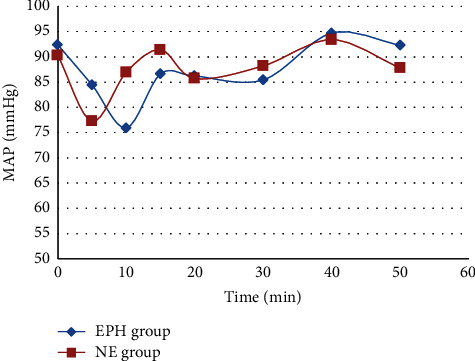
Comparison between norepinephrine and ephedrine groups according to mean arterial blood pressure change over time.

**Figure 2:**
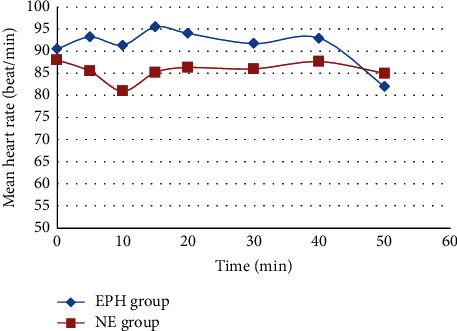
Comparison between norepinephrine and ephedrine groups according to mean heart rate change over time.

The NE group had a statistically significant higher MAP compared to the EPH group on the 10 and 15 min (*p* < 0.05) records, although the EPH group had a higher MAP on the first 5 min record (*p* < 0.05). After 20 min of spinal anaesthesia induction, there was no statistically significant difference in MAP between the groups until the end of the study period (*p* > 0.05) (Figure 1). Regarding the heart rate, the EPH group had a statistically significant higher heart rate throughout the procedure compared to the NE group (*p* < 0.05) (Figure 2).

**Figure 3:**
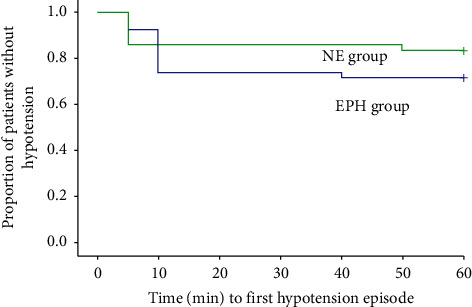
A Kaplan–Meier curve depicting the change in mean arterial blood pressure between the groups over time.

The Kaplan–Meier survival analysis graph presented in the following section to compare the proportion of hypotension episodes between the groups over time shows there was no significant difference between the groups with the log-rank test (*p*=0.245) (Figure 3).

### 3.2. Number of Boluses of Vasopressor and Atropine Needed

The NE group required a lower bolus number of vasopressors compared to the EPH group to maintain the blood pressure. The required bolus number of atropine between the two groups was not different (*p*=0.46). Two patients from EPH and five patients from the NE group developed bradycardia (HR < 60), and the heart rate returned to normal with a single dose of 0.5 mg atropine (Table 2).

### 3.3. Fetal Apgar Score

Regarding the fetal Apgar score, the Apgar scores of all newborns at 1 and 5 min were above seven [7]. There was no statistically significant difference between the two groups at both 1 and 5 min (Table 3).

### 3.4. Intraoperative Maternal Complication

Significant differences regarding maternal complications (nausea and vomiting) between the NE and EPH groups were not detected (nausea, *p*=0.21 and vomiting, *p*=0.092).

## 4. Discussion

This study evaluated the effectiveness of a prophylactic bolus dose of EPH and NE to maintain the MAP of a parturient above 80% of their baseline value during spinal anaesthesia for CS. According to this study, the NE group maintained MAP with fewer episodes of hypotension and a lower number of NE requirements compared to the EPH group. Moreover, the NE group maintained a lower frequency of the heart rate compared with the EPH group.

In a randomized, double-blinded study comparing the MAP and HR among patients who received 5 mg EPH and NE 10 *μ*g (i. v.) bolus, MAP was maintained, and HR was lower in the NE group [13]. This result is consistent with the present study, although the dose of EPH and NE used was not similar. In another prospective double-blinded trial, the incidence of maternal hypotension and the number of vasopressor doses used were lower in the NE group compared with EPH (*p*=0.02, 0.005, and 0.01, respectively) when measured 5 min after spinal anaesthesia [12]. This result coincides with the present study results.

According to the study conducted among 120 pregnant women undergoing elective CS under spinal anaesthesia, parturients were randomly divided into “group E” for ephedrine and “group N” for norepinephrine, with 60 females in each group. Group E received 10 mg of i. v. ephedrine, and Group N received 16 *μ*g of norepinephrine as hypotension prophylaxis at the time of intrathecal block. Compared with group E, a parturient who took N maintained blood pressure above 80% of their baseline value [14]. This finding is consistent with our study; however, a statistically significant frequency of bradycardia is reported from the norepinephrine group.

Another study compared the efficacy of intermittent bolus administration of EPH versus NE for treatment of hypotension after spinal anaesthesia. The results revealed that EPH group 16 (35.6%) and NE group 21 (46.7%) of patients did not need additional boluses of the vasopressor, but the difference was not statistically significant [15]. This result is different from ours that the NE group had a statistically significant lower number of boluses than the EPH group. This difference may be attributed to the difference in the bolus dose of the vasopressor used (EPH, 5 mg iv; NE group) and the study population in which the study was conducted on patients having spinal anaesthesia for lower limb orthopaedic surgery.

A study conducted to assess the efficacy and safety of bolus administration of NE versus EPH for the maintenance of systolic blood pressure during spinal anaesthesia in coronary artery disease patients undergoing knee arthroscopy showed that efficacy was found in 20 (40%) patients of group EPH and in 40 (80%) patients of group NE. The results were highly significant (*p* < 0.001) [16]. These results coincide with the results of our study, in spite of the different NE (5 *μ*g iv) and EPH (5 mg iv) bolus doses used as well as the different study population.

Women undergoing elective CS were administered NE at 4 *μ*g/minute or EPH at 4 mg/minute immediately after spinal anaesthesia. Infusion of 4 *μ*g/minute NE presented fewer episodes of tachycardia and a lower HR compared to the EPH infusion group, as well as better foetal outcome. In terms of umbilical artery blood gas results, the NE group had a higher base excess (BE) and a lower lactate level than the EPH group (both *p* < 0.001) [17]. Our study also demonstrated a lower heart rate for the NE group, although foetal outcome was similar between the groups with an Apgar score > 7 for all newborns.

A prospective, double-blind study compared different intermittent NE bolus doses of 3, 4, 5, 6, 7, or 8 g to determine the effective dose for a patient undergoing caesarean section. The use of intermittent IV NE boluses to prevent spinal-induced hypotension in elective CS seems feasible and was not observed to be associated with adverse outcomes. Practically, they suggest an ED90 dose of 6 g and recommend further work which is warranted to elucidate the comparative effects of intermittent IV bolus doses of phenylephrine and NE, in terms of efficacy and safety [18].

## 5. Conclusion

NE can be used as a substitute to EPH to maintain the blood pressure of a pregnant mother undergoing elective CS under spinal anaesthesia without adverse effects on the mother and baby.

## Figures and Tables

**Table 1:** Demographic and intraoperative data.

Variables	Group EPH (*n* = 42)	Group NE (*n* = 42)	*p* value
Age, yr	28.7 ± 4.19	27.43 ± 4.8	0.196
BMI, (kg/m^2^)	22.48 ± 2.27	22.03 ± 2.01	0.35
Haemoglobin, (g/dl)	13.44 ± 0.93	13.3 ± 1.02	0.31
Gravida	3 (1)	3 (2)	0.32
Para	2 (2)	2 (1)	0.2
Skin incision to delivery, (min)	12 (5)	12 (6)	0.95
Blood loss, (ml)	400 (250)	400 (300)	0.49
Fluid infused, (ml)	2250 (1500)	2000 (1000)	0.16
Oxytocin, (IU)	20 (10)	20 (10)	0.35
Baseline MAP, (mm/hg)	92.43 ± 10.4	90.3 ± 10.1	0.34
Baseline HR, (beat/min)	81.23 ± 11.24	80.9 ± 10.15	0.88
Duration of surgery, (min)	55 (20)	52.5 (20)	0.86

BMI = body mass index; MAP = mean arterial pressure; HR = heart rate; independent sample *t*-test; Mann–Whitney *u* test; *p* value >0.05.

**Table 2:** The number of boluses of the vasopressor used.

	Group EPH (*n* = 42)	Group NE (*n* = 42)	*p* value
The number of boluses of vasopressor	2.5 (2)	1.5 (1)	0.001^*∗*^

Group EPH stands for ephedrine, and group NE stands for norepinephrine. Median (IQR) values, Mann–Whitney *u* test, and ^*∗*^*p* < value 0.05.

**Table 3:** The table shows the difference in Apgar scores between the groups at the 1st and 5th minute.

Fetal Apgar score	Group EPH (*n* = 42)	Group NE (*n* = 42)	*p* value
At 1 min	8 (1)	8 (2)	0.91
At 5 min	9 (2)	9 (1)	0.624

Group EPH stands for ephedrine, and group NE stands for norepinephrine. Values are median (IQR), Mann–Whitney *u* test; ^*∗*^*p* < value 0.05.

## Data Availability

The data used to support the findings of the study can be obtained from the corresponding author upon reasonable request.

## Additional Points

*Limitations*. Since this was a cohort study, the confounders might not have been sufficiently controlled.
